# Supplementary material for: LC-MS profiling and cytotoxic activity of Angiopteris helferiana against HepG2 cell line: Molecular insight to investigate anticancer agent
Source: PLoS One. 2024 Dec 31;19(12):e0309797. doi: 10.1371/journal.pone.0309797 (PMC11687663; doi:10.1371/journal.pone.0309797)

**LC-MS Profiling and Cytotoxic Activity of** ***Angiopteris helferiana* Against HepG2 Cell Line: Molecular Insight to Investigate Anticancer Agent**

Bipindra Pandey^1^, Shankar Thapa^1*^, Mahalakshmi Suresha Biradar^2^, Jaya Bahadur Ghale^3^, Pramod Kharel^4^, Prabhat Kumar Jha^5^, Ram Kishor Yadav^5^, Sujan Dawadi^6^

^1^Department of Pharmacy, Madan Bhandari Academy of Health Sciences, Hetauda, Nepal

^2^Department of Pharmaceutical Chemistry, Al-Ameen College of Pharmacy, Bengaluru, India

^3^Karnali College of Health Science, Purbanchal University, Nepal

^4^National Academy for Medical Science, Purbanchal University, Nepal

^5^School of Health and Allied Science, Pokhara University, Pokhara, Nepal

^6^Asian College for Advance Studies, Purbanchal University, Lalitpur, Nepal

**Supplementary file**

**Table1S. MS condition: Triple Quadrupole (QqQ) MSMS.**

| **ES Source** | |
| --- | --- |
| Capillary (kV) | 3.45 |
| Cone (V) | 33 |
| Extractor(V) | 3.0 |
| **Temperature** | |
| Source Temp | 110 |
| Desolvation Temp | 350 |
| **Gas Flow** | |
| Desolvation (L/hr.) | 750 |
| Cone (L/hr.) | 50 |
| Scan Time | 0.2 Sec |
| **Software:** | |
| **For HPLC** | LC-Solution 1.25 SP4 |
| **For MS** | MassLynx V4.1SCN805 |

**Table 2S. Sample preparation and analysis.**

| **Time** | **Solution A** | **Solution B** |
| --- | --- | --- |
| 0.01 | 85 | 15 |
| 6.00 | 25 | 75 |
| 8.00 | 25 | 75 |
| 11.0 | 85 | 15 |
| 15.0 | 85 | 15 |
| 15.01 | Controller | Stop |


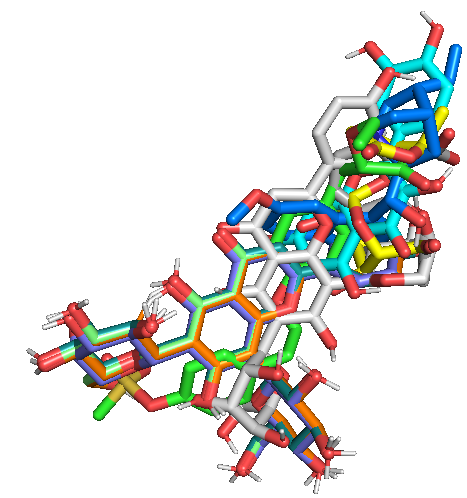


**Fig 1S**. Phytoconstituents alignment (**B1** to **B17)** with native co-crystal ligand to evaluate RMSD value.


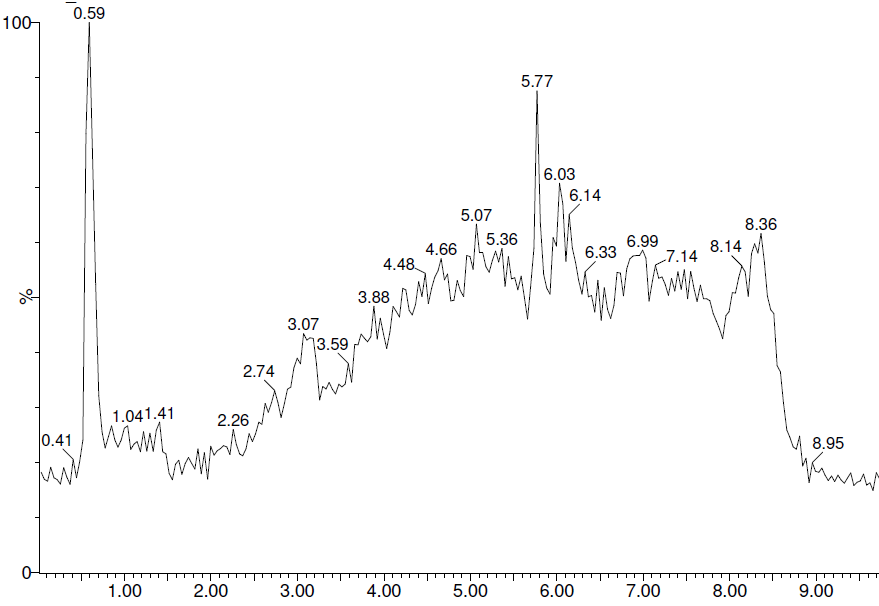


**Fig 2S**. Chromatogram of plant extract *Angiopteris helferiana*.


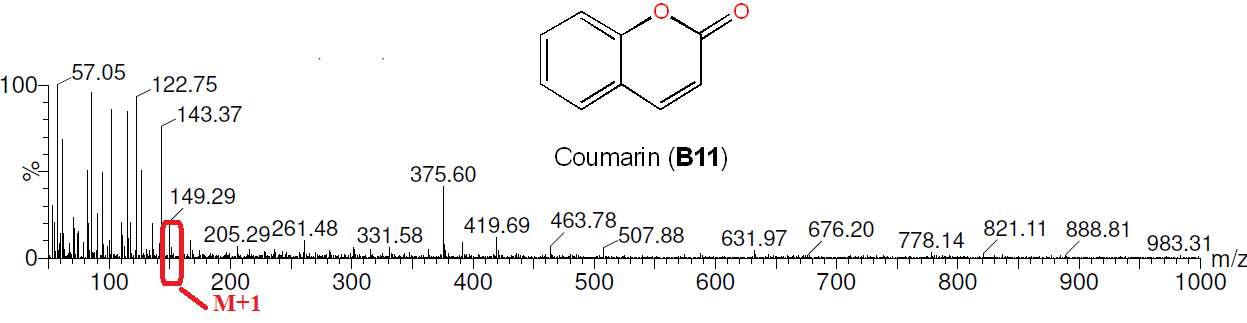


**Fig 3S.** Mass spectrum of Coumarin (**B11**) from *Angiopteris helferiana*.


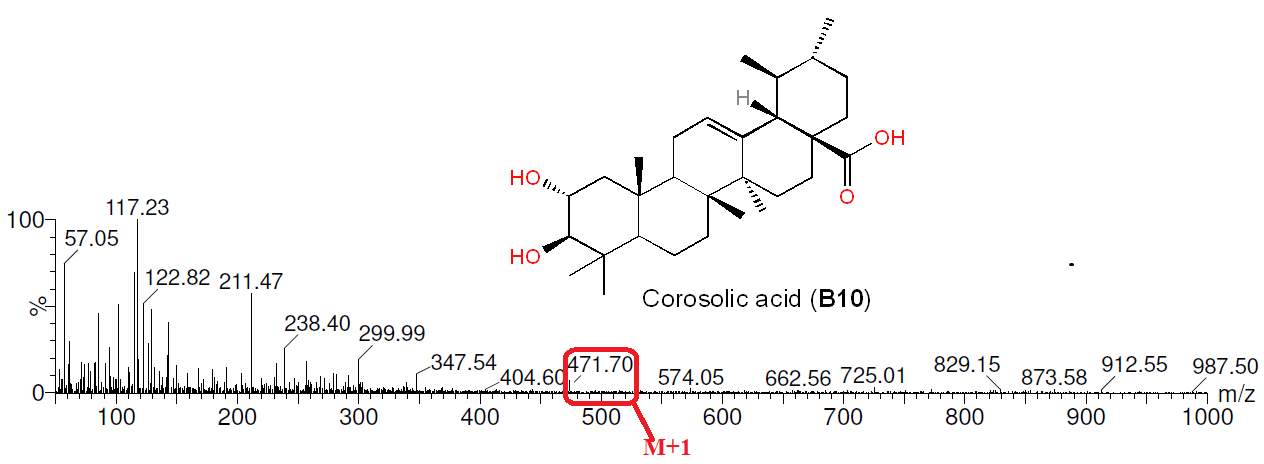


**Fig 4S.** Mass spectrum of Corosolic acid (**B10**) from *Angiopteris helferiana*.


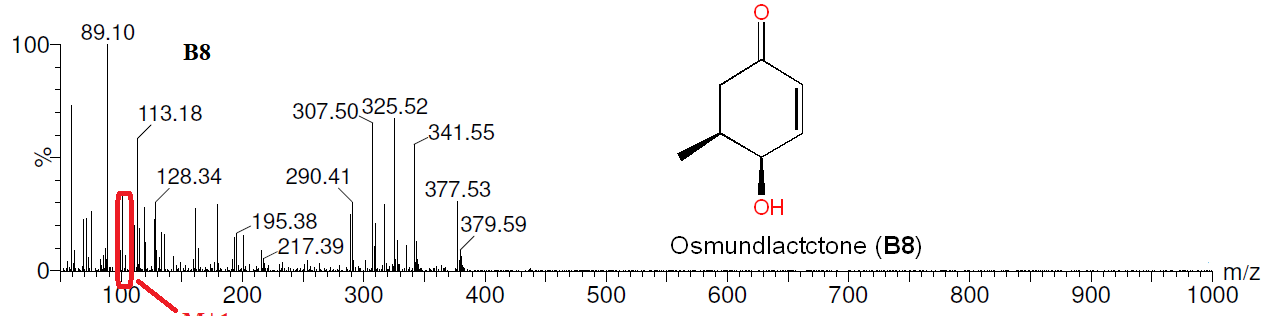


**Fig 5S**. Mass spectrum of Osmundlactotone (**B8**) from *Angiopteris helferiana*.


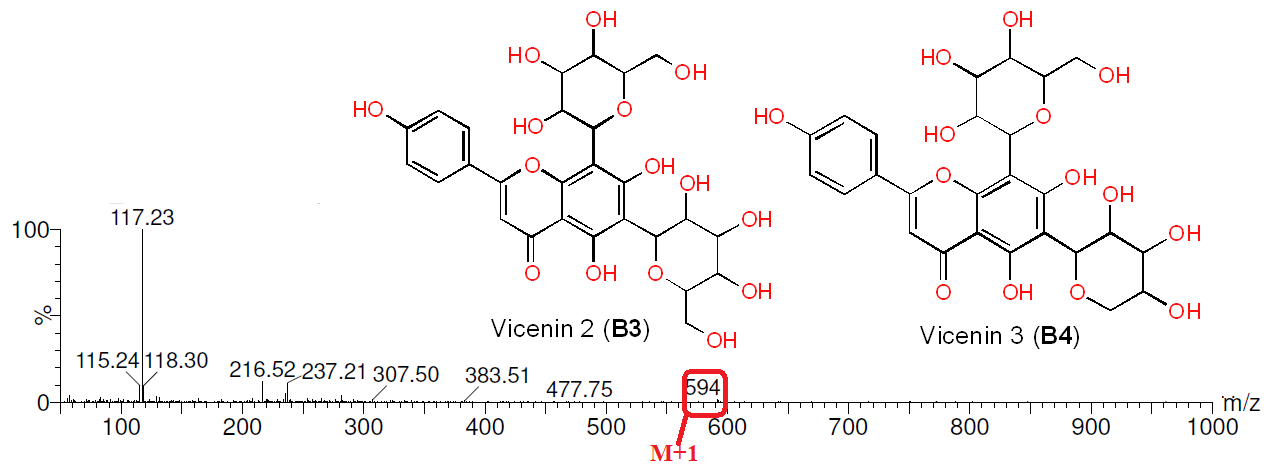


**Fig 6S.** Mass spectrum of Viecenin 1(**B3**) and Vicenin 3 (**B4**) from *Angiopteris helferiana*.


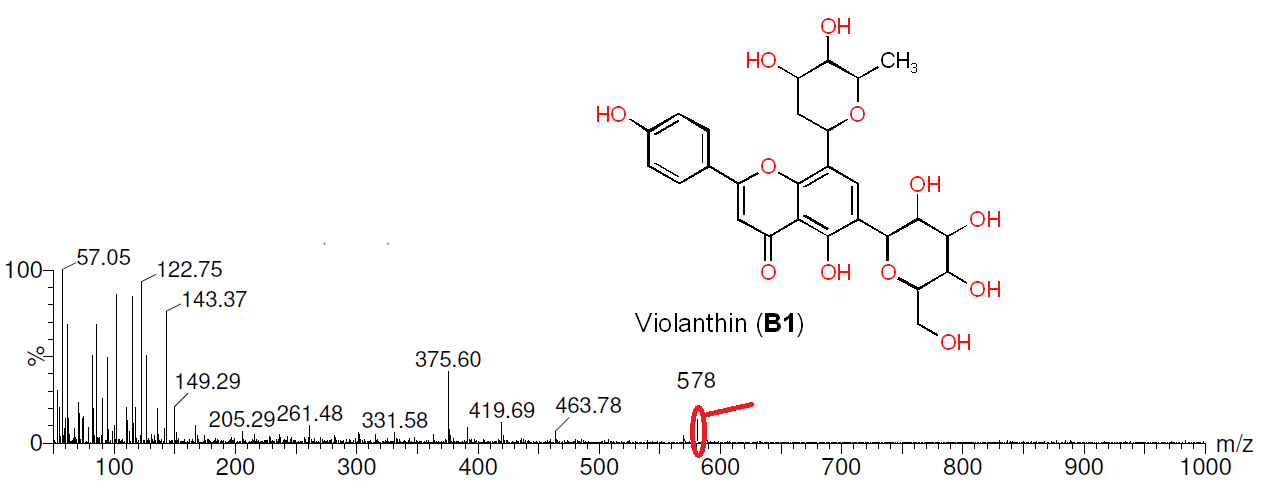


**Fig 7S**. Mass spectrum of Violanthin (**B1**) from *Angiopteris helferiana*.


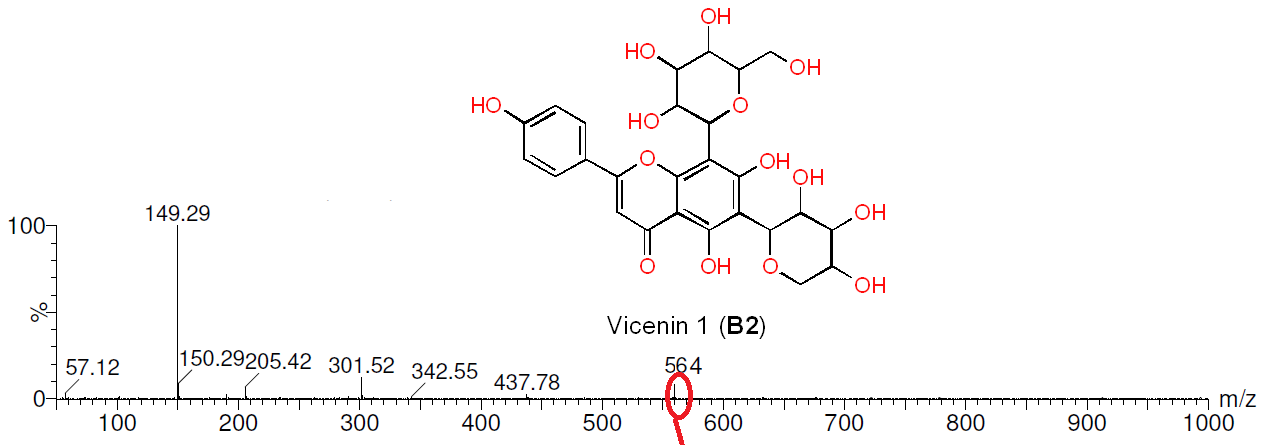


**Fig 8S.** Mass spectrum of Vicenin (**B2**) from *Angiopteris helferiana*.


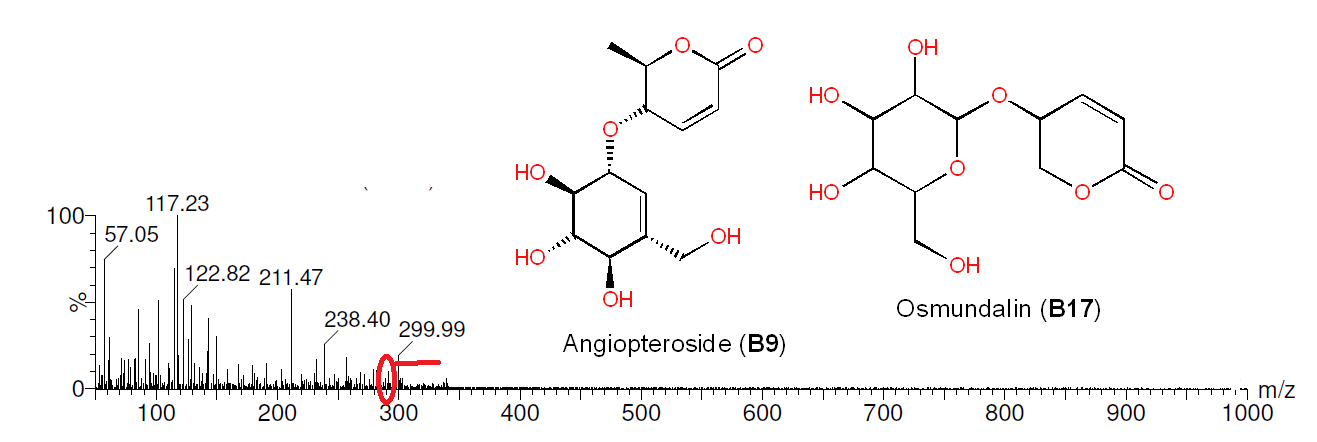


**Fig 9S.** Mass spectrum of Angiopteroside (**B9**) from *Angiopteris helferiana*.


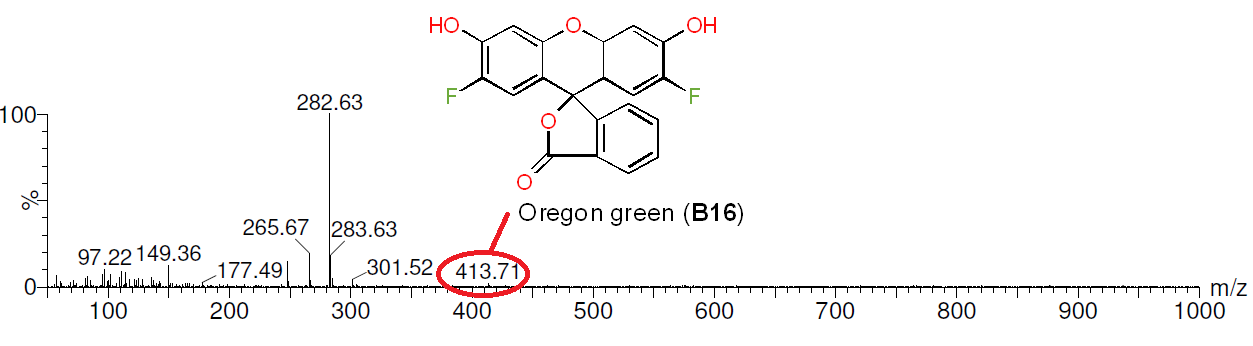


**Fig 10S.** Mass spectrum of Oregon green (**B16**) from *Angiopteris helferiana*.


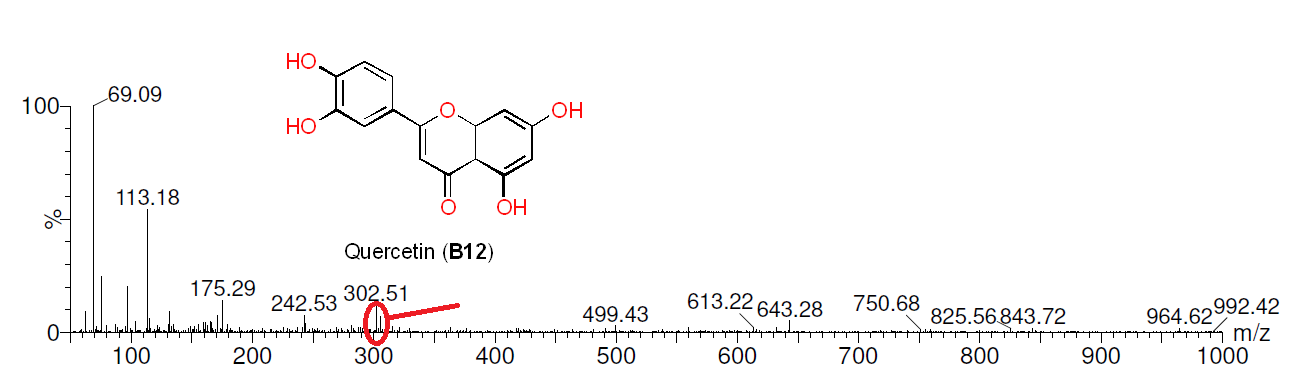


**Fig 11S.** Mass spectrum of Quercetin (**B12**) from *Angiopteris helferiana*.


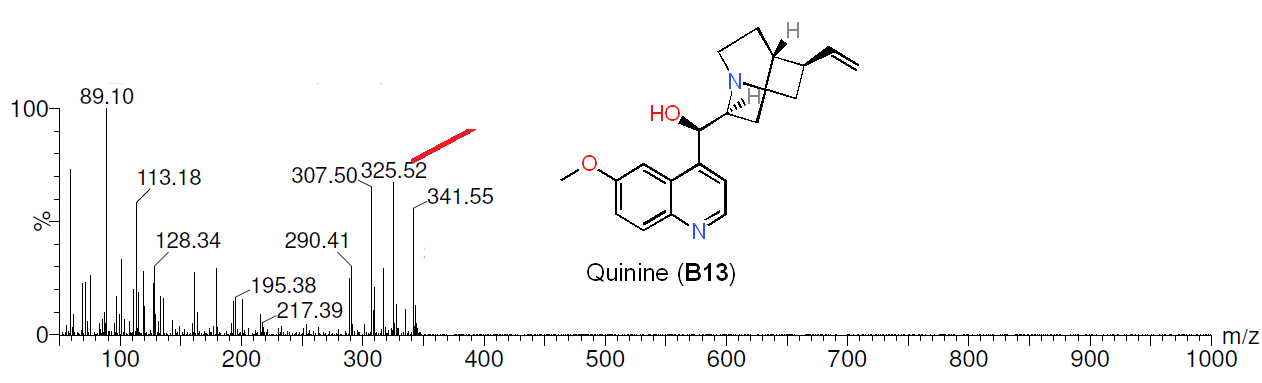


**Fig 12S.** Mass spectrum of Quinine (**B13**) from *Angiopteris helferiana*.


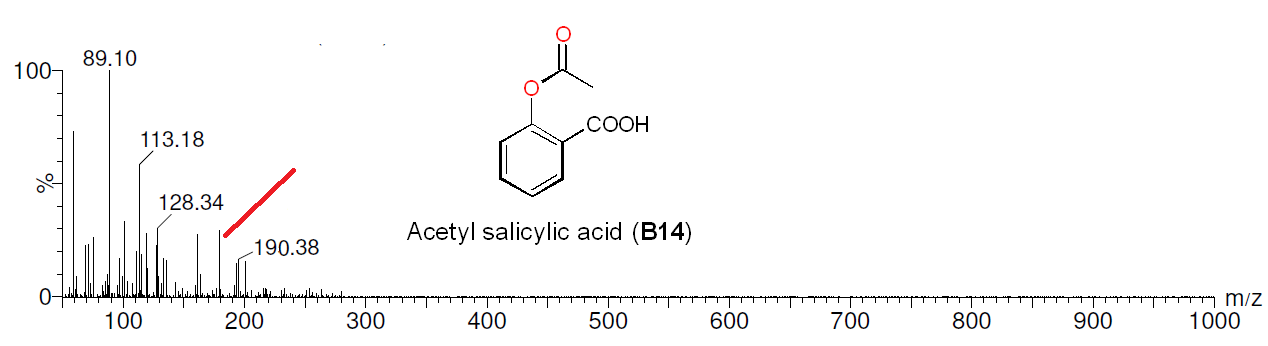


**Fig 13S.** Mass spectrum of Acetyl salicylic acid (**B14**) from *Angiopteris helferiana*.


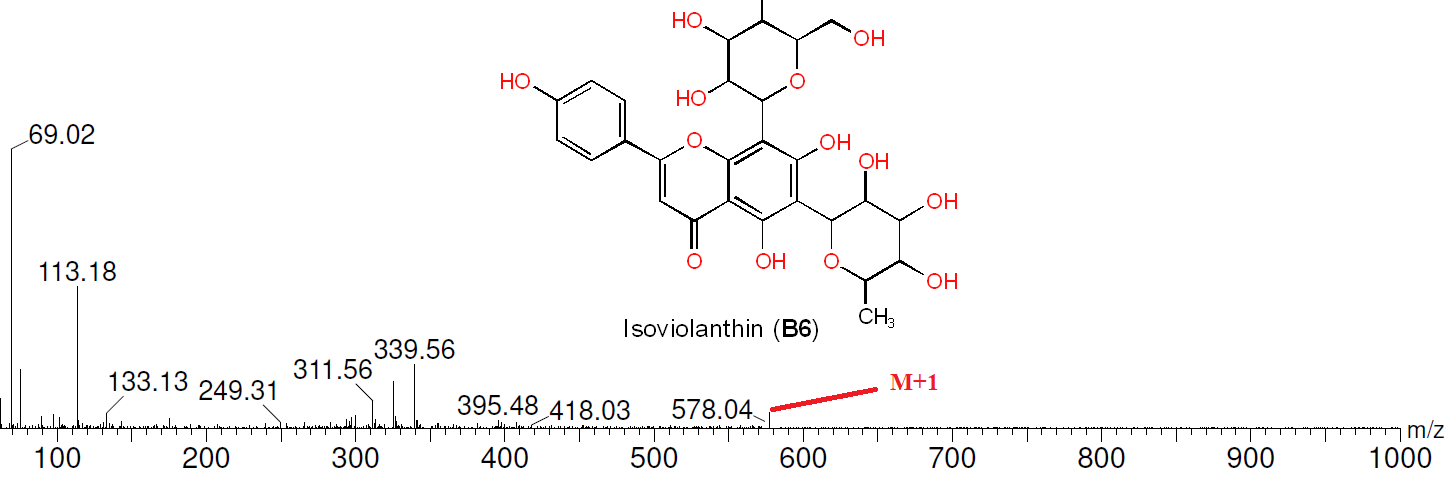


**Fig 14S.** Mass spectrum of Isoviolanthin (**B6**) from *Angiopteris helferiana*.


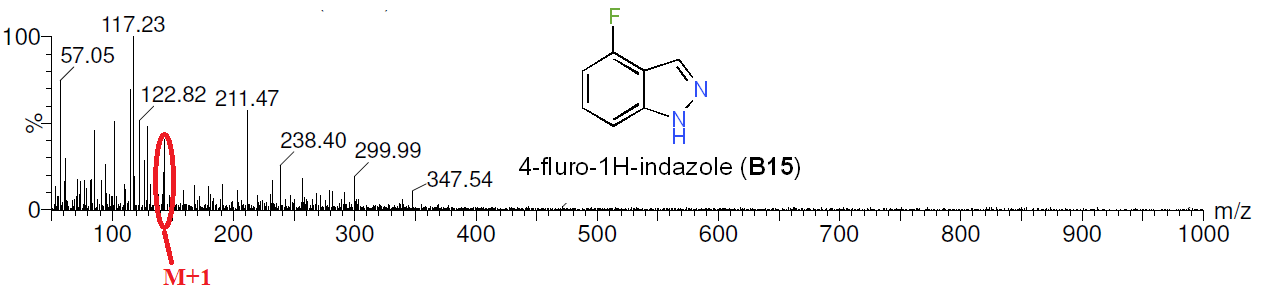


**Fig 15S.** Mass spectrum of 4-fluro-1H-indazole (**B15**) from *Angiopteris helferiana*.


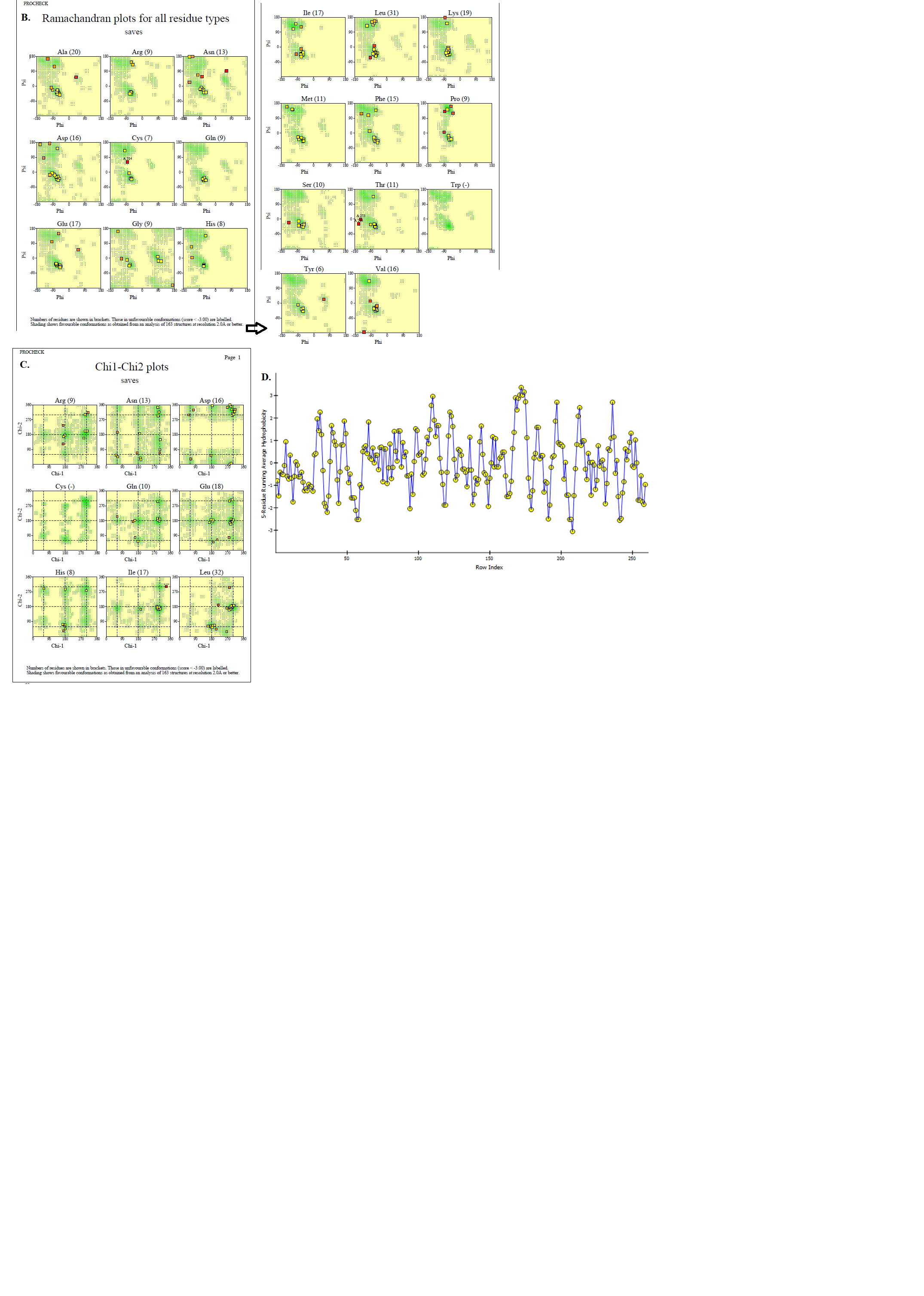


**Fig 16S.** Protein validation. **B.** Ramachandran plots for all residues. **C.**Chi1-Chi2 plots. **D.** Hydrophobicity of PPAR-δ (PDBID: 1I7G).


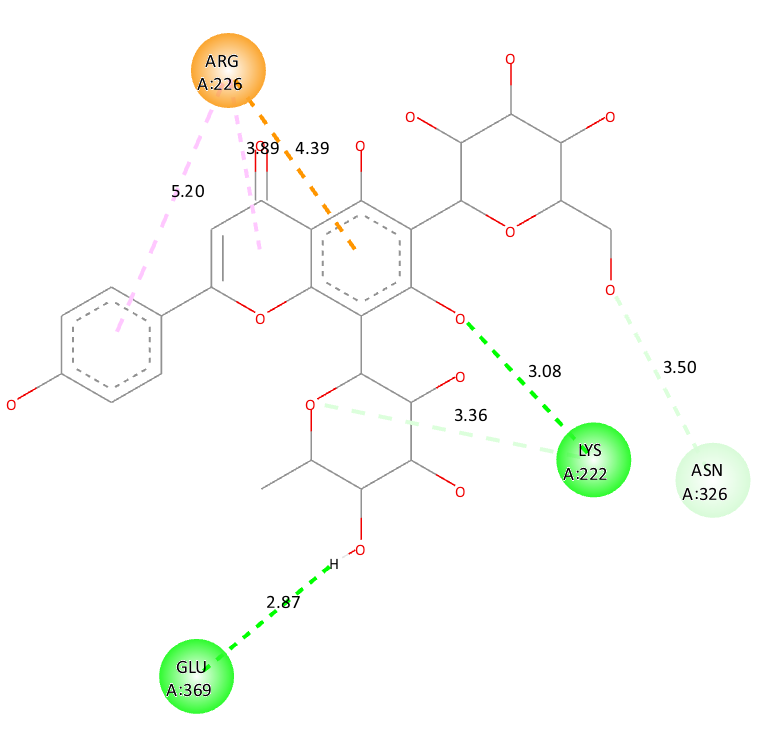


**Fig 17S.** 2D molecular interaction of compound **B1** with PPAR-δ (PDBID: 1I7G).


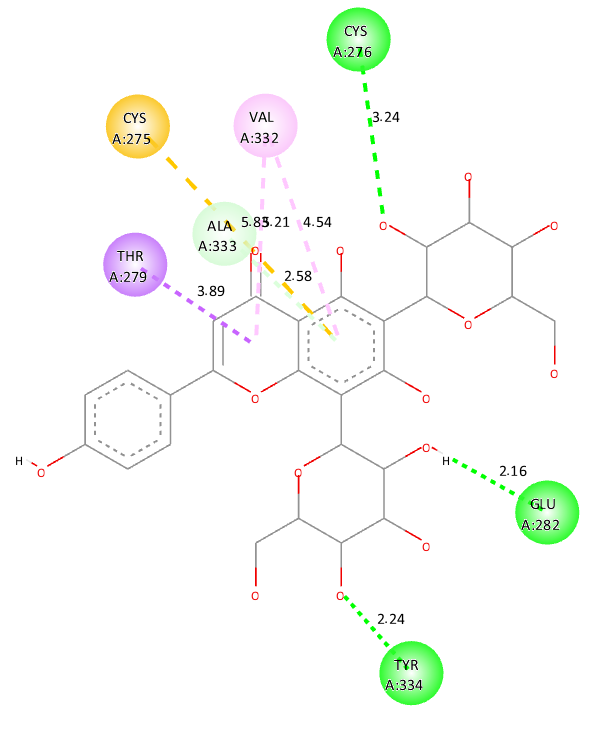


**Fig 18S.** 2D molecular interaction of compound **B3** with PPAR-δ (PDBID: 1I7G).


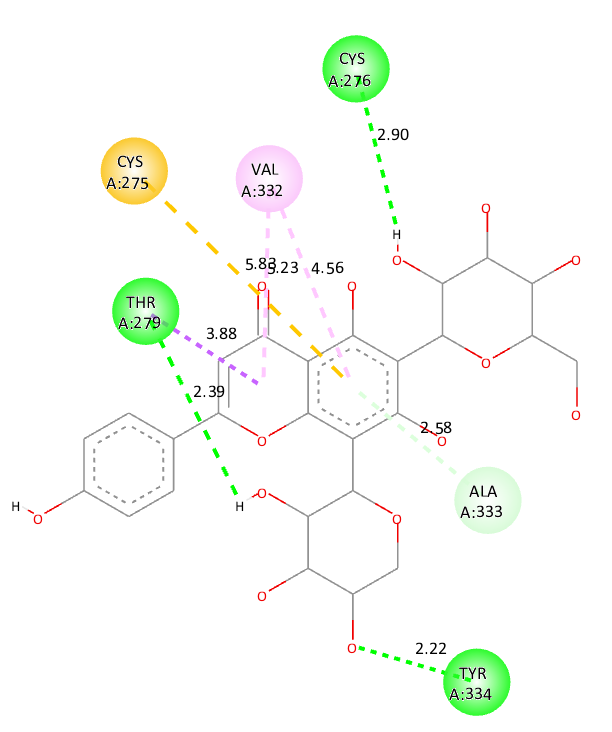


**Fig 19S.** 2D molecular interaction of compound **B4** with PPAR-δ (PDBID: 1I7G).


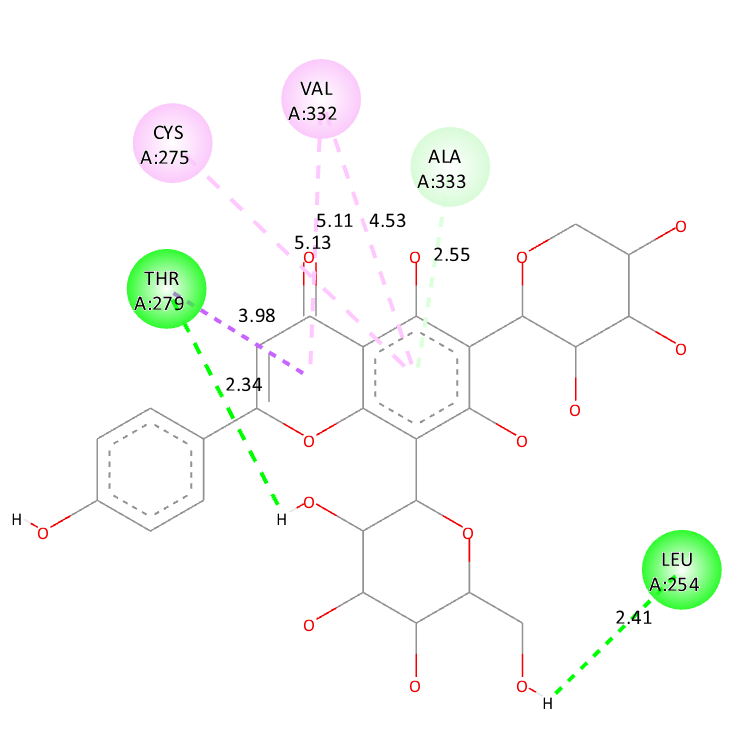


**Fig 20S.** 2D molecular interaction of compound **B5** with PPAR-δ (PDBID: 1I7G).


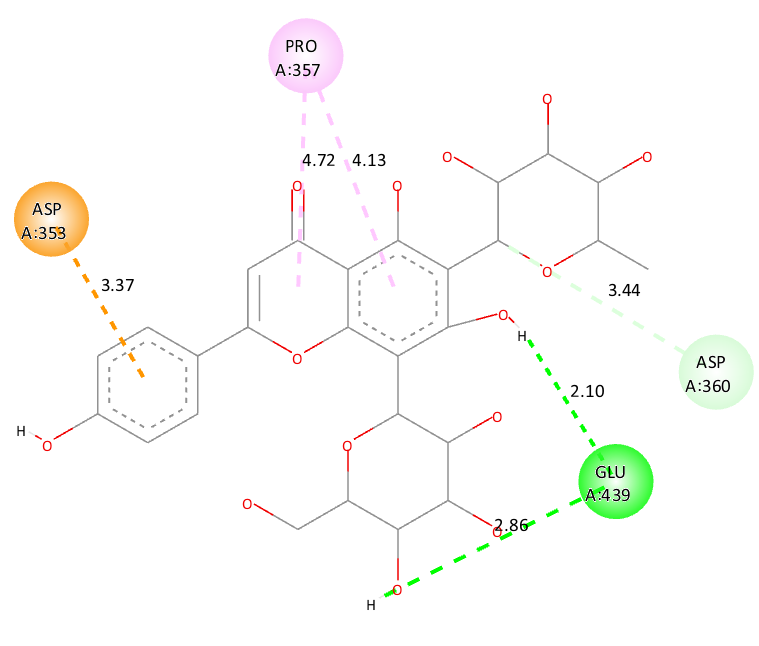


**Fig 21S.** 2D molecular interaction of compound **B6** with PPAR-δ (PDBID: 1I7G).
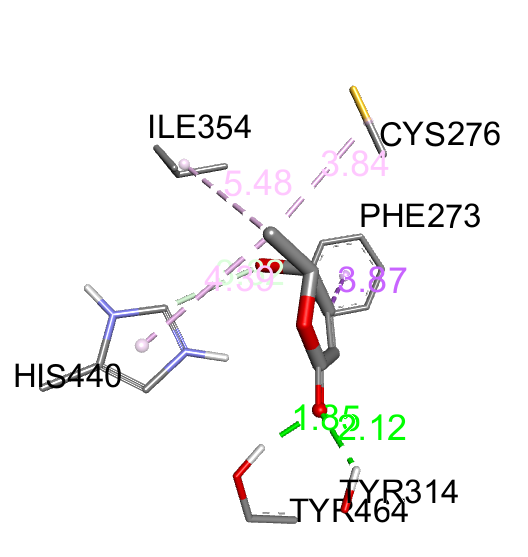


**Fig 22S.** 3D molecular interaction of compound **B8** with PPAR-δ (PDBID: 1I7G).


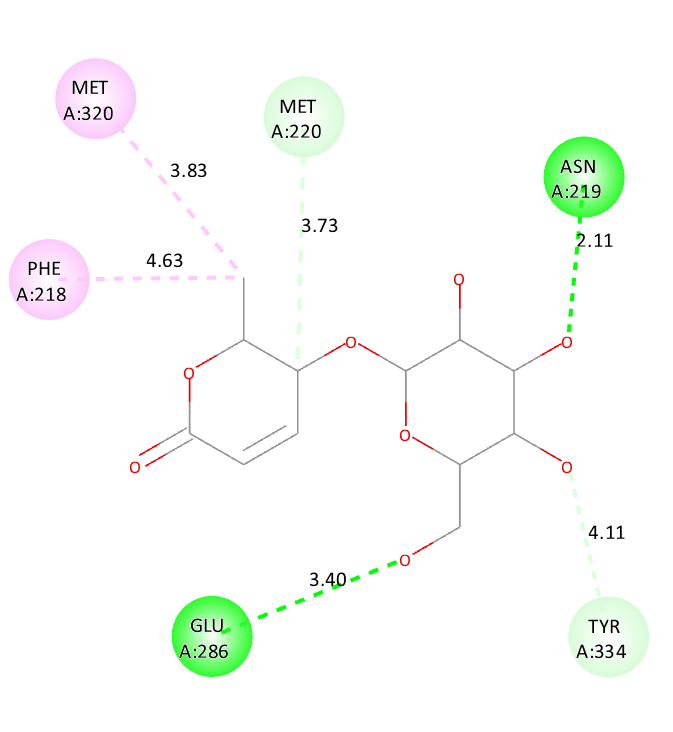


**Fig 23S.** 2D molecular interaction of compound B9 with PPAR-δ (PDBID: 1I7G).


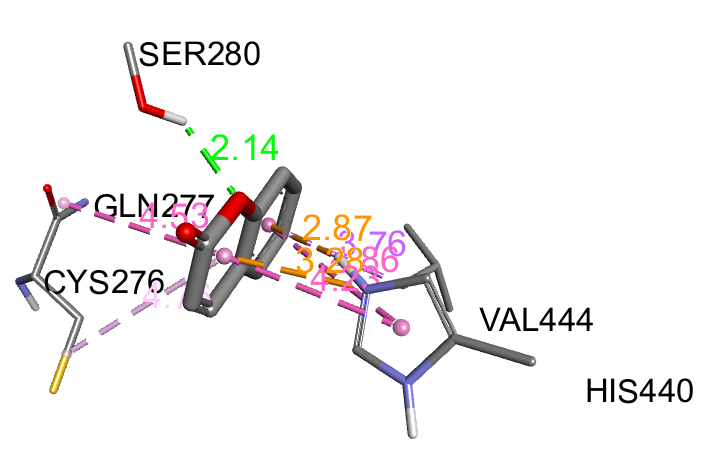


**Fig 24S.** 3D molecular interaction of compound **B11** with PPAR-δ (PDBID: 1I7G).


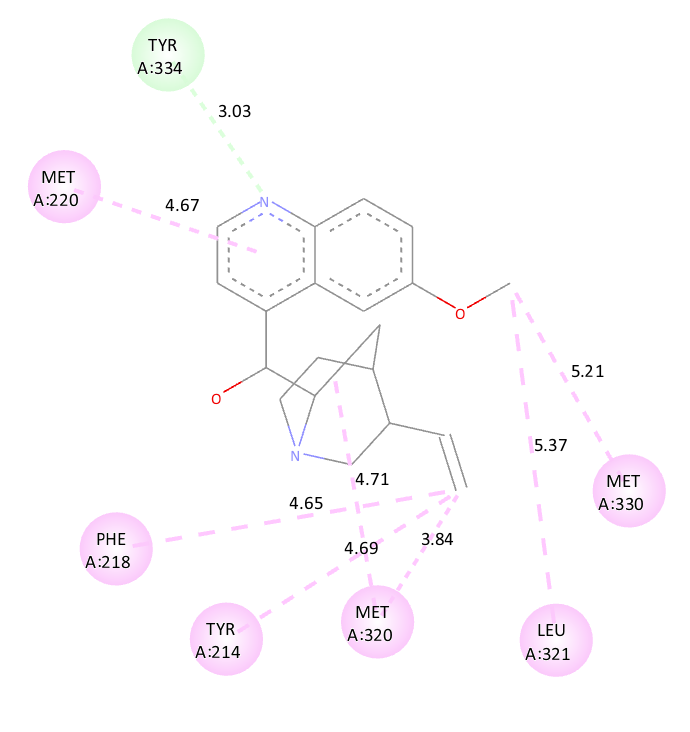


**Fig 25S.** 2D molecular interaction of compound **B13** with PPAR-δ (PDBID: 1I7G).


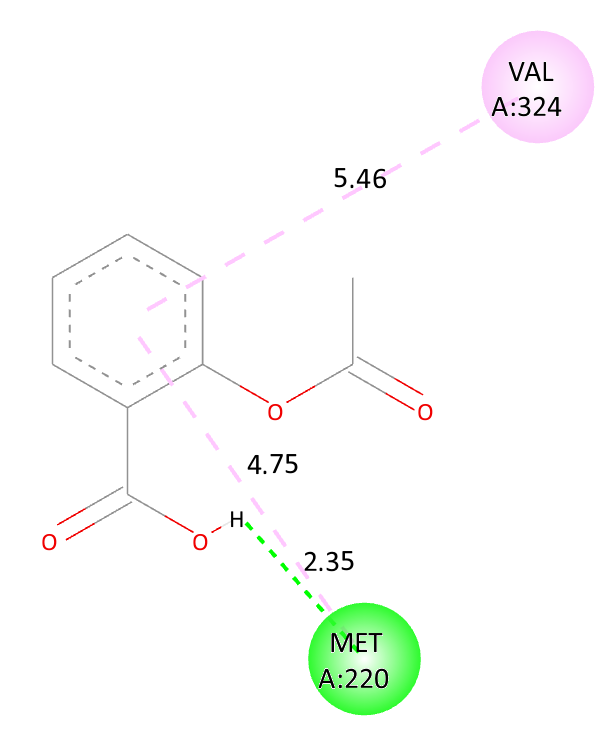


**Fig 26S.** 2D molecular interaction of compound **B14** with PPAR-δ (PDBID: 1I7G).


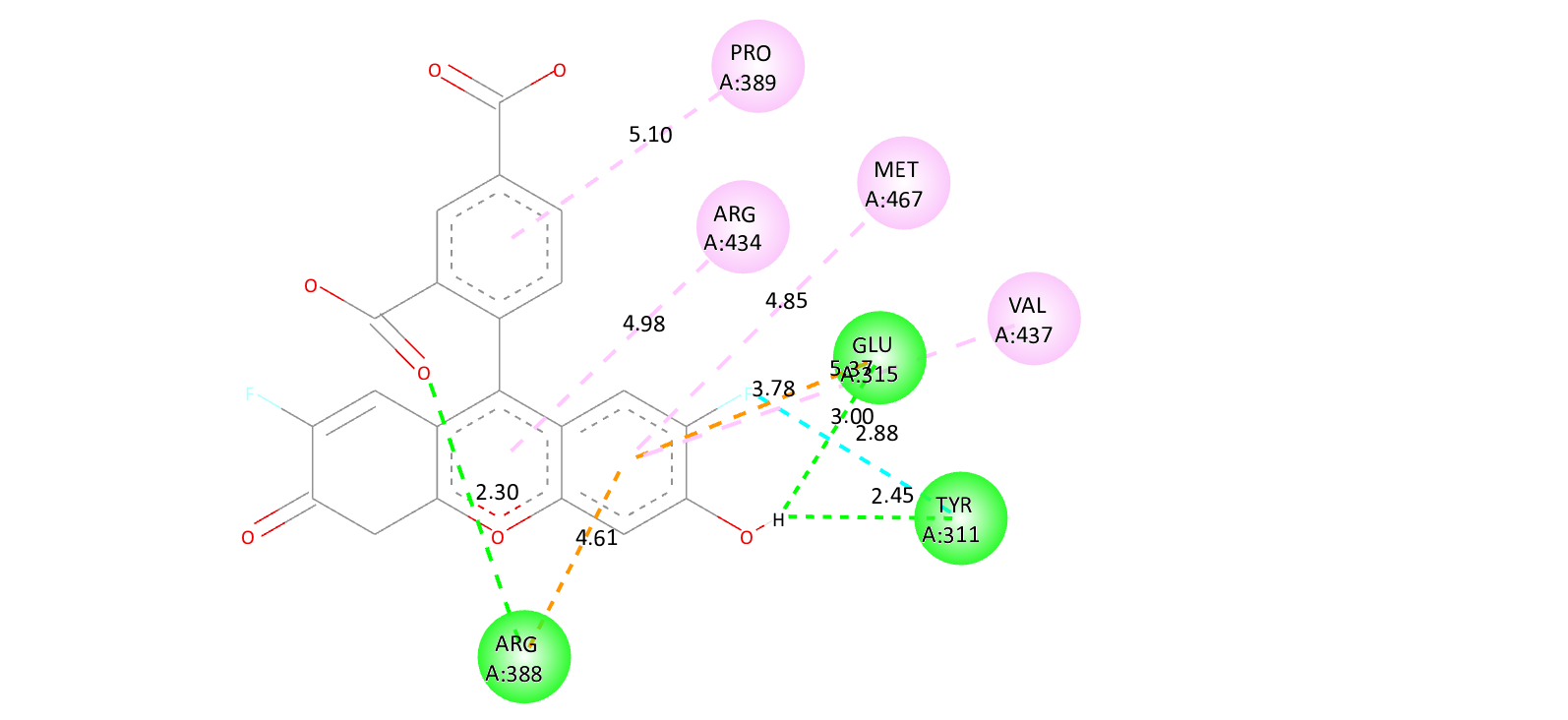


**Fig 27S.** 2D molecular interaction of compound **B16** with PPAR-δ (PDBID: 1I7G).


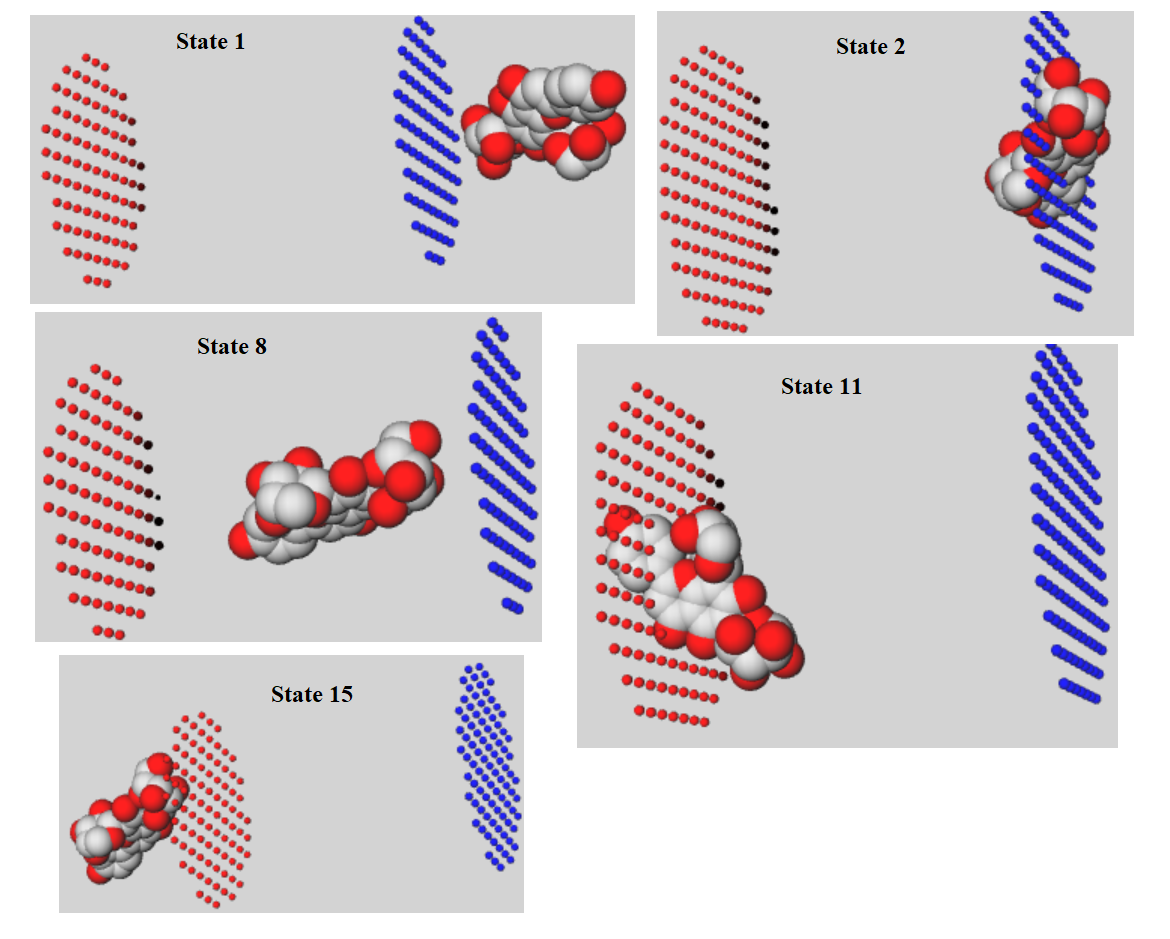


**Fig 28S.** Passive translocation mechanism of compound **B7**.


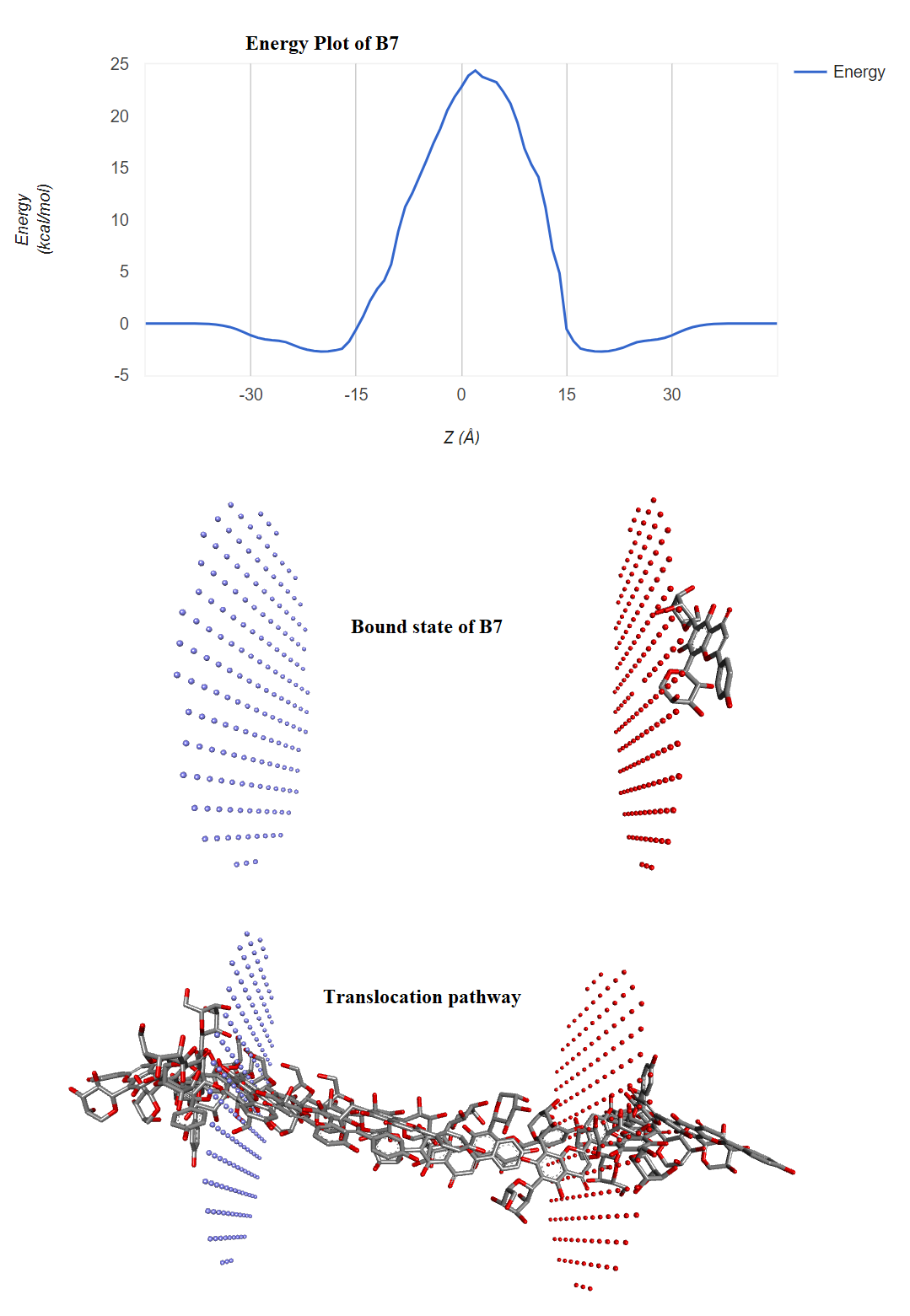


**Fig 29S.** Energy plot and mechanism of action of compound **B7** at bound state and translocation pathway.

**Table 3S. Energy plot and membrane binding energy of** **B7**.


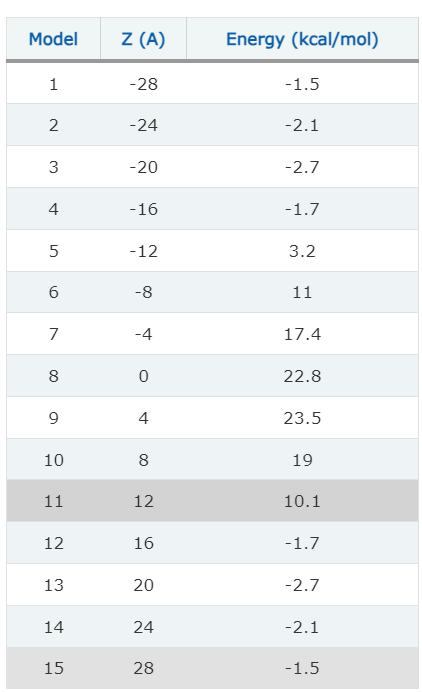


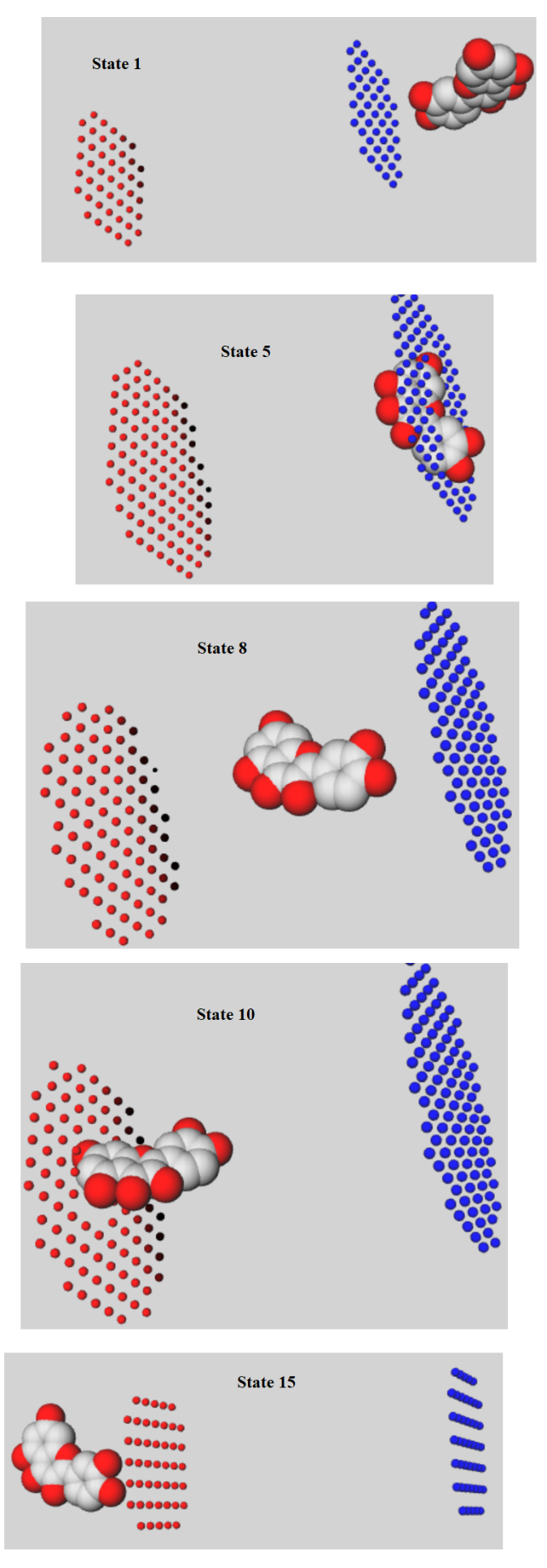


**Fig 30S.** Passive translocation mechanism of compound **B12**.


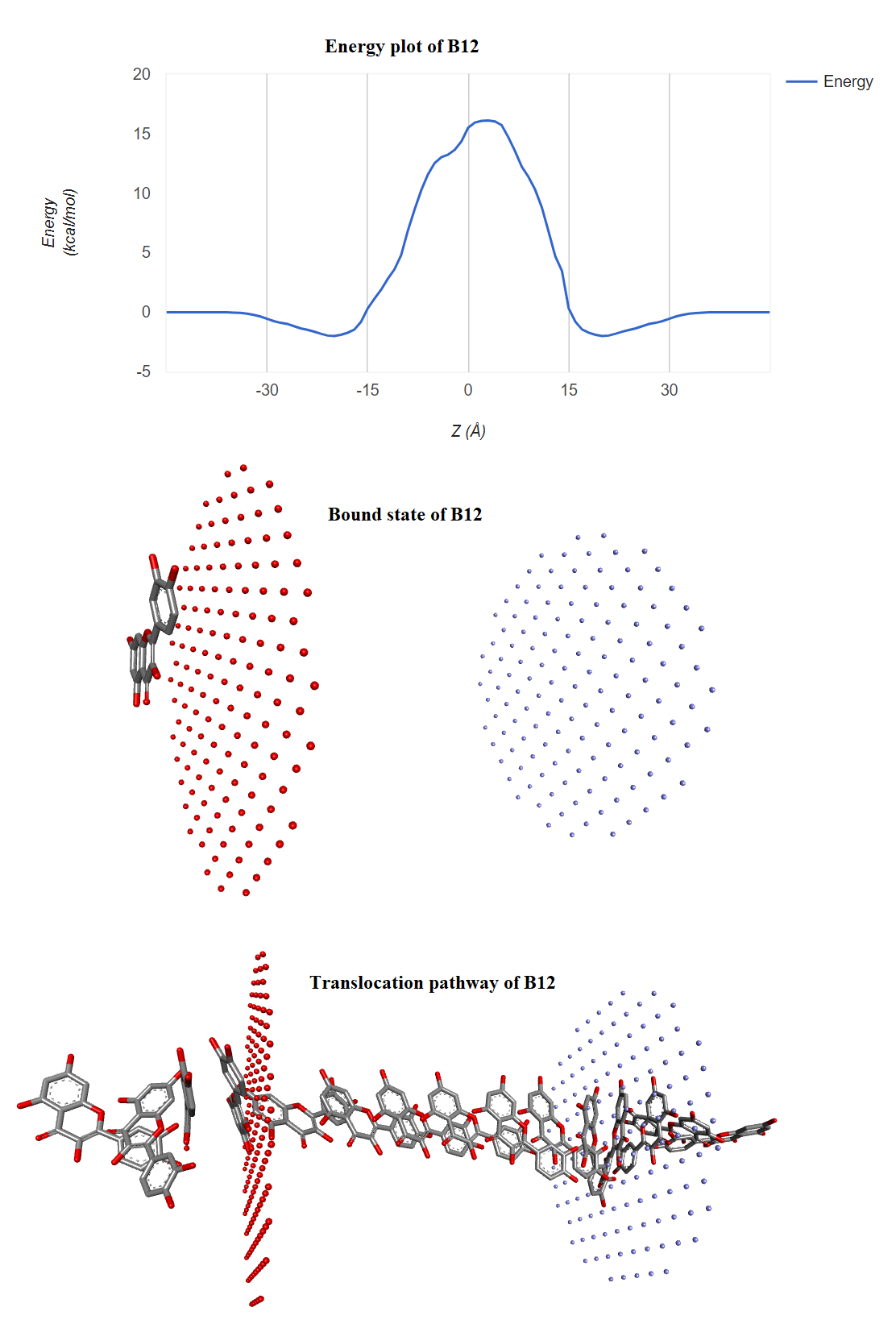


**Fig 31S.** Energy plot and mechanism of action of compound **B12** at bound state and translocation pathway.

**Table 4S. Energy plot and membrane binding energy of** **B12**.


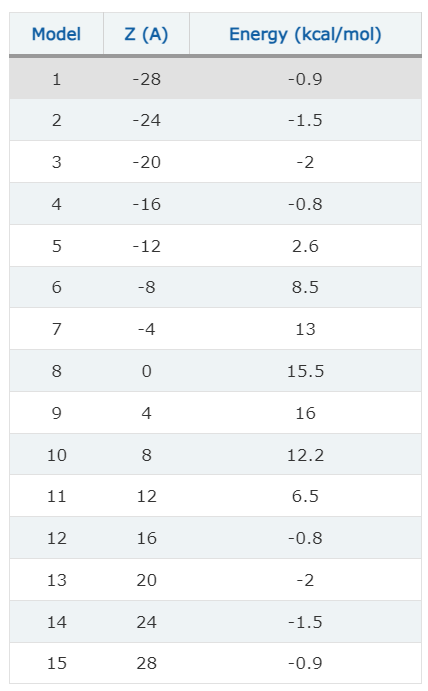

Supplement: S1 File — (DOCX) [file pone.0309797.s001.docx]
