# Supplementary material for: LC-MS profiling and cytotoxic activity of Angiopteris helferiana against HepG2 cell line: Molecular insight to investigate anticancer agent
Source: PLoS One. 2024 Dec 31;19(12):e0309797. doi: 10.1371/journal.pone.0309797 (PMC11687663; doi:10.1371/journal.pone.0309797)
Supplement: Attachment — Submitted filename: Response to Reviwers.docx [file pone.0309797.s003.docx]

May 16, 2024

Dear editor and reviewers,

Thank you very much for your comments and suggestions. We appreciate reviewers and editors for their effort to provide insightful comments and suggestions in our manuscript. We strongly believe that the revised manuscript has substantially improved after addressing the comments and suggestions. Here, we have highlighted our point-by-point comments and the accompanying adjustments to the manuscript. The revised sentences are highlighted by yellow color in the manuscript.

We look forward to your kind consideration.

Shankar Thapa

On behalf of all co-authors

Corresponding author

**Reviewer 1**

**Comment 1:** The work is oriented towards the anticancer effect of exttract of Angiopteris helferiana (A. helferiana) C. Presl is a large 88 fleshy fern. However it is traditionaly used in many diseases, I have to reaise a question on what base the authors selected the anticancer against one line of cells? is it indicative results to get the results against HepG2 Cell only?Also, the activity due to the extract however the authors recorded the activity is due to very well-known compounds like quercetion where there are a lot of reports regardong this compound as anticancer and up till now not used, So, extensive study on these componds is consider a waste of time and effeort.

I think the authors have to work in purified fraction preceded with activity guided fractionation to get standartized active simple fraction could be applied in pharmaceutical market.

-table 1- column 10 it is coumarin not falavonoid because coumarin is 9 carbons mostly and flavonoid is 15 carbons

Please revise the content very well.

discussion need more information to get the conclusion in right way.

**Response:** We thank reviewer for valuable suggestion. We agree with reviewer and we have incorporated basis of selection for anti-cancer activity against HepG2 cell line. We also want to highlight the fact that, there is no valuable research found for this particular plant. We have added the rationale on the basis of other fern belonging to the family Marattiaceae. Please refer page 4, line 101-109. Furthermore, the discussion part is also revised with proper information.

**Reviewer 2**

**Comment 1:**  The authors must compare the effect of cyto-toxic on normal cell line like RPE1 or BJ1

**Response:** We would like to thank for the comments. Reviewer suggested to compare the cyto-toxic effect on normal cell like RPE1 or BJ1. We understand the comments, if we compare with normal cell line, it will provide the data on toxicity report. However, limited research on the plant makes us difficult to find the relevant article. As of our best knowledge, cytotoxic effect of *Angiopteris helferiana* is not explored yet. For this research, our aim is to explore the cytotoxic effect of plant on HepG2 cell line through the mean of computational and in-vitro study.

**Comment 2:** The IC 50 of 5-fluorouracil is too high. The time of contact between extract or drug with the cell is not mentioned.

**Response:** Thank you for pointing this out. We have revised the IC50 (5.0 μg/mL) value of 5-flurouracil in the manuscript with proper citation. The time of contact was 24 hours. We have added this time in manuscript. Please kindly refers to methodology section.

**Reviewer 3**

**Comment 1:** Results and discussion: lines111/112: peak at 2927cm-1 confirms the presence of phenolic group? The band 2927cm-1 is attributed the aliphatic C-H stretching, revise the value again. Lines 116/117: 1392 cm-1and 1269 cm-1indicates the involvement of stretching vibration of different aliphatic alkyl groups.?? The given 1392 and 1269cm-1 values are not due to C-H stretching? Please change the assignment values again.

**Response:** Thank you for pointing this out. We believe that, this was tyo error and now we have revised the sentence as “Moreover, the presence of sharp peak at 2927cm-1 confirms the presence of aliphatic C-H group” and another sentence also revised as “A peak at 1392 cm⁻1 was likely due to the C-H bending vibrations from methyl groups or potentially symmetric stretching from a nitro group. Similarly, a peak at 1269 cm⁻1 was likely due to the C-O stretching vibrations from an ether group or deformation from a nitro group.” Please kindly refer to page 5, line 121, 126-129.

**Comment 2**: 2.4 Molecular Docking: line: 174, A. helferiana extract) when bound to a target molecule (human PPAR-δ receptor). Why did the author choose Humn PPAR-δ as the enzyme-protein target for A.helferiana more than alternative binding targets? Like EGFR and VEGFR? HDACs, etc. Provide reasonable explanations. Furthermore, did the author do docking binding studies with other targets than PPAR-δ, and verified that PPAR-δ is the best for interaction according to binding free energy (Kcal/mol) and RMSD (Ẩ)? The author provided a thorough description of the interactions between the secondary metabolites and the target protein, including the sorts of interactions.

**Response:** Thank you for your valuable suggestion. We have provided the reason for selection of PPAR protein as target. Our point of selection for protein was “ PPAR-δ is crucial in the pathophysiology of HCC along with the role in metabolic process[59]. This is the novel and most promising receptor for the development of anti-cancer agents. Various clinical observation reported that, the epidermal growth factor receptor (EGRF) is suspectable to develop resistance against drugs[60]. But, PPARs are ligand-activated transcription factors that are part of the nuclear hormone receptor superfamily, playing crucial roles in regulating energy balance, cell differentiation, proliferation, apoptosis, and inflammation. Initial studies connected PPARs to the development of cancer, and currently, PPARs are associated with various solid organ cancers, including those of the breast, ovary, prostate, bladder, stomach, colon, and lungs. Therefore, we choose PPAR- δ for our research as receptor[61], [62].” We believe that, this explanation will validate the research questions. Moreover, we have provided the reason for choosing that particular protein, PDBID: 1I7G. Please refer, page, line 472-474.

**Comment 3:** Analysis of passive membrane permeability and translocation pathways, line:328: compounds (Menthol carbonates) described by Mollazadeh et al, cite reference number for this author.

**Response:** Thank you for pointing this out. We have added the proper citation on that line.

**Comments 4:** HepG2 cell line study

Lines: 338-340: This screening was conducted to validate the in-silico molecular docking results. Interestingly, the extract showed the significant cytotoxic activity having IC50 value of 236.93 μg/ml (Table 7 and Figure 9). This result indicates the potential activity of extract towards HepG2 Human liver cancer cell line.>>> The IC50 value of 236.93µg/ML for the extract is low-moderate and not notable

NOTE: An extract is defined to be highly active if it has IC50 < 10 µg/ml, active when the IC50 is between 10 µg/ml and 100 µg/ml, moderately active if the IC50 is between 100µg/ml and 500µg/ml, and low activity if the IC50 is ˃ 500 µg/ml. Bahadori, M H., Azari, Z., Zaminy, A., Dabirian, S., Mehrdad, S M., & Kondori, B J. (2021, March 31). Anti-proliferative and apoptotic effects of hull-less pumpkin extract on human papillary thyroid carcinoma cell line. 54(1), 104-111. <https://doi.org/10.5115/acb.20.22>.

The author should test the cytotoxicity of the extract on a normal cell line, and then calculate the selectivity index (SI) (IC50 normal cells / IC50 cancer cells).

Line: 342: The reference (35): The drug 5-Flurouracil, IC50= (435.59 μg/ml (35)), and Table 7:>>> This reference specifies the IC50 against the HCT116 cell line?? NOT against the HEPG2 cancer cell line; correct the statement and conclusion, which do not match the HEPG2 cell line that you employed.

**Response:** We are extremely thankful for this valuable comments. We have revised the results and added your suggestion in the manuscript. Moreover, we have revised the IC50 value of reference drugs with proper citation of HepG2 cell line. We have corrected the conclusion as per revision. The standard data for IC50 value of extract was also incorporated in manuscript. Please kindly refer page 17 and 18. For SI, due to resources limitation we are unable to conduct the test. We also believe that, it will improve the quality of paper, but we are from Nepal, where limited resource make us difficult to conduct such test.
